# Supplementary material for: The complete structure of the human TFIIH core complex
Source: eLife. 2019 Mar 12;8:e44771. doi: 10.7554/eLife.44771 (PMC6422496; doi:10.7554/eLife.44771)
Supplement: Supplementary file 1. — All datasets were acquired on Gatan K2 Summit direct electron detectors mounted in 300 kV-electron microscopes with three-condenser type electron optics. The high rejection rate for the data collected without VPP was due to poorer CTF resolution estimates compared to the VPP data, likely because of the use of a low-base Titan with a less stable side entry holder for most of these data. Abbreviations: TEM, transmission electron microscope; VPP, volta phase plate; Σ, sum. [file elife-44771-supp1.docx]

| **Data set #** | **TEM** | **VPP** | **Pixel  size (Å)** | **Total  dose  (e^-^/Å^2^)** | **Frames** | **K2 acquisition mode** | **Total  movies** | **Movies  retained** | **Particles  after  initial 3D sorting** | **Particles in final map** | **Data reported** |
| --- | --- | --- | --- | --- | --- | --- | --- | --- | --- | --- | --- |
| 1 | Titan  KRIOS | Yes | 1.15 | 50 | 33 | Super-resolution | 3,270 | 2,047 | 154,298 | 27,165 | This study |
| 2 | Titan  KRIOS | Yes | 1.15 | 50 | 33 | Super-resolution | 3,190 | 1,804 | 134,654 | 23,034 | This study |
| 3 | Titan  KRIOS | Yes | 1.15 | 50 | 33 | Super-resolution | 4,804 | 3,269 | 191,830 | 18,365 | This study |
| 4 | Titan  KRIOS | Yes | 1.15 | 50 | 33 | Super-resolution | 3,283 | 2,709 | 107,390 | 32,008 | This study |
| 5 | Titan  KRIOS | Yes | 1.15 | 50 | 33 | Super-resolution | 4,958 | 2,857 | 85,582 | 22,717 | This study |
| 6 | Titan  KRIOS | Yes | 1.15 | 50 | 50 | Super-resolution | 1,932 | 1,631 | 113,001 | 15,370 | This study |
| 7 | Titan  KRIOS | No | 1.32 | 40 | 30 | Super-resolution | 2,719 | 476 | 12,788 | - | This study |
| 8 | Titan | No | 1.32 | 40 | 30 | Counting | 1,734 | 215 | 3,942 | - | Greber et al. 2017 |
| 9 | Titan | No | 1.32 | 40 | 30 | Counting | 2,694 | 262 | 4,617 | - | Greber et al. 2017 |
| 10 | Titan | No | 1.32 | 40 | 30 | Counting | 2,593 | 653 | 19,642 | - | Greber et al. 2017 |
| Σ |  |  |  |  |  |  | 31,177 | 15,923 | 827,744 | 138,659 |  |
