# Supplementary material for: The complete structure of the human TFIIH core complex
Source: eLife. 2019 Mar 12;8:e44771. doi: 10.7554/eLife.44771 (PMC6422496; doi:10.7554/eLife.44771)
Supplement: Supplementary file 2. [file elife-44771-supp2.docx]

| **Protein** | **Chain ID** | **Size^*^  (aa)** | **Modeled residues** | **Sequence accession code** | **Alternative names** | **Comments** |
| --- | --- | --- | --- | --- | --- | --- |
| XPB | A | 782 | 34-204,  248-730 | P19447 | ERCC3 | Residues 248-265 built as poly- alanines and deposited as UNK. |
| XPD | B | 760 | 1-760 | P18074 | ERCC2 | 4Fe4S-cluster: SF4,  residue 1000; residues 292-318 built as poly-alanines and deposited as UNK. |
| p62 | C | 548 | 107-173,  183-321,  346-547 | P32780 | GTF2H1 | BSD1 domain docked and refined with reference restraints; residues 148-173, 183-189, 233-296, 365-371,390-395, 443-452,480-520, and 540-547 built as poly-alanines and deposited as UNK. |
| p52 | D | 462 | 7-458 | Q92759 | GTF2H4 |  |
| p44 | E | 395 | 16-274,  281-387 | Q13888 | GTF2H2 | Zn^2+^: residues 401, 402, 403; residues 16-50, 260-274, and 281-286 built as poly-alanines and deposited as UNK. |
| p34 | F | 308 | 8-72,  95-292 | Q13889 | GTF2H3 | Zn^2+^: residue 401. |
| p8 | G | 71 | 2-67 | Q6ZYL4 | GTF2H5, TTDA | - |
| CDK7 | - | 346 | - | P50613 | MO15 | Not visualized. |
| Cyclin H | - | 323 | - | P51946 | CCNH | Not visualized. |
| MAT1 | H | 309 | 1-210 | P51948 | MNAT1 | Zn^2+^: residues 400, 401; N-terminal RING domain docked and refined with reference restraints. |

^*^ According to Uniprot (http://www.uniprot.org).

**^†^** Unassigned sequences were initially modeled and refined as poly-alanine and deposited as UNK.
