# Supplementary material for: The complete structure of the human TFIIH core complex
Source: eLife. 2019 Mar 12;8:e44771. doi: 10.7554/eLife.44771 (PMC6422496; doi:10.7554/eLife.44771)
Supplement: Supplementary file 3. [file elife-44771-supp3.docx]

| **Data collection** | **Datasets 1-6** |
| --- | --- |
| Microscope | Titan KRIOS |
| Stage type | Autoloader |
| Voltage (kV) | 300 |
| Detector | K2 Summit |
| Energy filter | GIF Quantum |
| Other | Volta phase plate |
| Pixel size (Å) | 1.15 |
| Defocus range (μm) | 0.3-1.2 |
| Electron dose (e^-^/A^2^) | 50 |
| **Reconstruction** | **EMD-0452** |
| Software | RELION 3 |
| Particles | 138,659 |
| Box size (pixels) | 256 x 256 x 256 |
| Accuracy rotations (°) | 1.4 |
| Accuracy translations (pixels) | 0.8 |
| Map resolution (Å) | 3.7 |
| Map sharpening B-factor (Å^2^) | -142 |
| **Coordinate refinement** |  |
| Software | PHENIX |
| Algorithm | REAL SPACE REFINE |
| Box size (Å) | 294.4 x 294.4 x 294.4 |
| Resolution cutoff (Å) | 3.7 |
| FSC_model-vs-map_=0.5 (Å) | 3.9 |
| **Model** | **PDB-6NMI** |
| Number of residues | 3185 |
| Protein | 3178 |
| Ligand (Fe_4_S_4_, Zn^2+^) | 7 |
| B-factors overall | 74.3 |
| Protein | 74.3 |
| Ligand (Fe_4_S_4_) | 58.4 |
| Ligand (Zn^2+^) | 140.9 |
| R.m.s. deviations |  |
| Bond lengths (Å) | 0.008 |
| Bond angles () | 1.080 |
| **Validation** |  |
| Molprobity score (percentile) | 1.9 (100^th^) |
| Molprobity clashscore (percentile) | 6.1 (100^th^) |
| Rotamer outliers (%) | 0.16 |
| C_β_ deviations (%) | 0 |
| Ramachandran plot |  |
| Favored (%) | 89.2 |
| Allowed (%) | 10.6 |
| Outliers (%) | 0.2 |
